# Case report: The origin of transmantle-like features

**DOI:** 10.3389/fradi.2022.927764

**Published:** 2022-07-29

**Authors:** Takeshi Matsuo, So Fujimoto, Takashi Komori, Yasuhiro Nakata

**Affiliations:** ^1^Department of Neurosurgery, Tokyo Metropolitan Neurological Hospital, Fuchu, Japan; ^2^Department of Laboratory Medicine and Pathology, Tokyo Metropolitan Neurological Hospital, Fuchu, Japan; ^3^Department of Neuroradiology, Tokyo Metropolitan Neurological Hospital, Fuchu, Japan

**Keywords:** transmantle sign, focal cortical dysplasia, corpora amylacea, the radial-oriented white matter band, tuberous sclerosis

## Abstract

The transmantle sign is considered to be a magnetic resonance imaging feature specific to patients with type II focal cortical dysplasia; however, this sign can be difficult to distinguish from other pathologies, such as a radial-oriented white matter band in tuberous sclerosis. Here, we report a case showing a high-intensity area on T2-weighted and fluid-attenuated inversion recovery images extending from the ventricle to the cortex associated with atypical histopathological findings containing corpora amylacea. This case demonstrates that some instances of transmantle signs may be due to corpora amylacea accumulation.

## Introduction

The transmantle sign is a magnetic resonance imaging (MRI) feature that is often seen in patients with type II focal cortical dysplasia (FCD). However, the origin of the transmantle sign is not well-known. Here, we consider transmantle-like features from the view of histopathological findings.

## Case description

The patient was a female in her fifties. She had experienced numbness and tingling sensation in the right upper limb during childhood. No medical treatment was administered at that time because no abnormalities were noted upon examination. About 10 years ago, she noticed involuntary twitching in her fingers and the corner of her mouth. Both abnormal movements appeared in her right side and lasted 20–30 s. She was diagnosed with epilepsy and treatment with antiepileptic drugs (AED) was initiated. Although several types of AEDs were prescribed, the seizures were intractable. Subsequently, the patient was referred to our hospital for surgical treatment.

The numbness in the right upper limb occurred daily, and mandibular twitching occurred weekly. Electroencephalography revealed focal spikes over the left frontoparietal lesion of the brain. Neuropsychological examination using the Wechsler Adult Intelligence Scale 4th Edition revealed that her full scale intelligence quotient was 109.

Written informed consent was obtained from the patient for publication of this case report and accompanying images.

## Image examination

**Figure 1:**
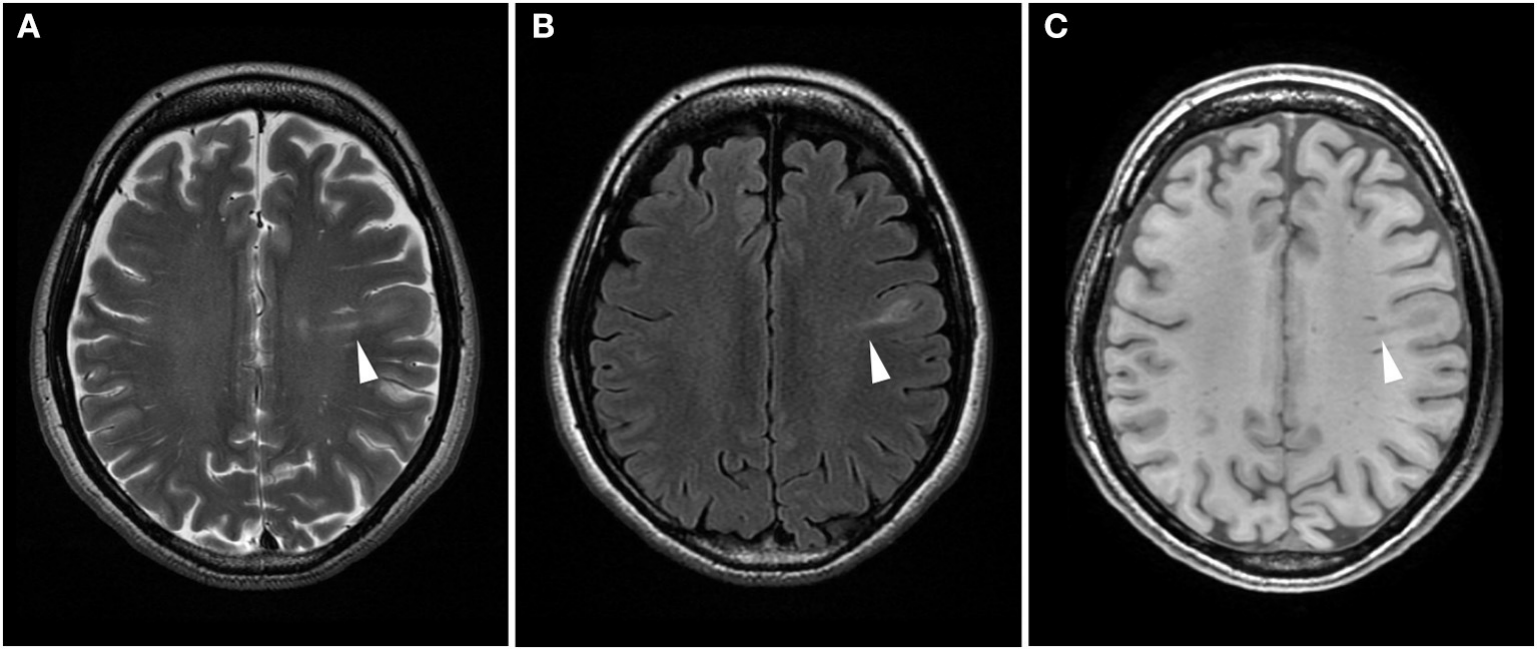
Difference in the signal intensity of a transmantle lesion across magnetic resonance imaging sequences. Axial T2-weighted **(A)** and fluid attenuated inversion recovery **(B)** images show high-intensity areas, while spoiled gradient recalled echo image **(C)** reveals low-intensity areas (arrowhead).

**Figure 2:**
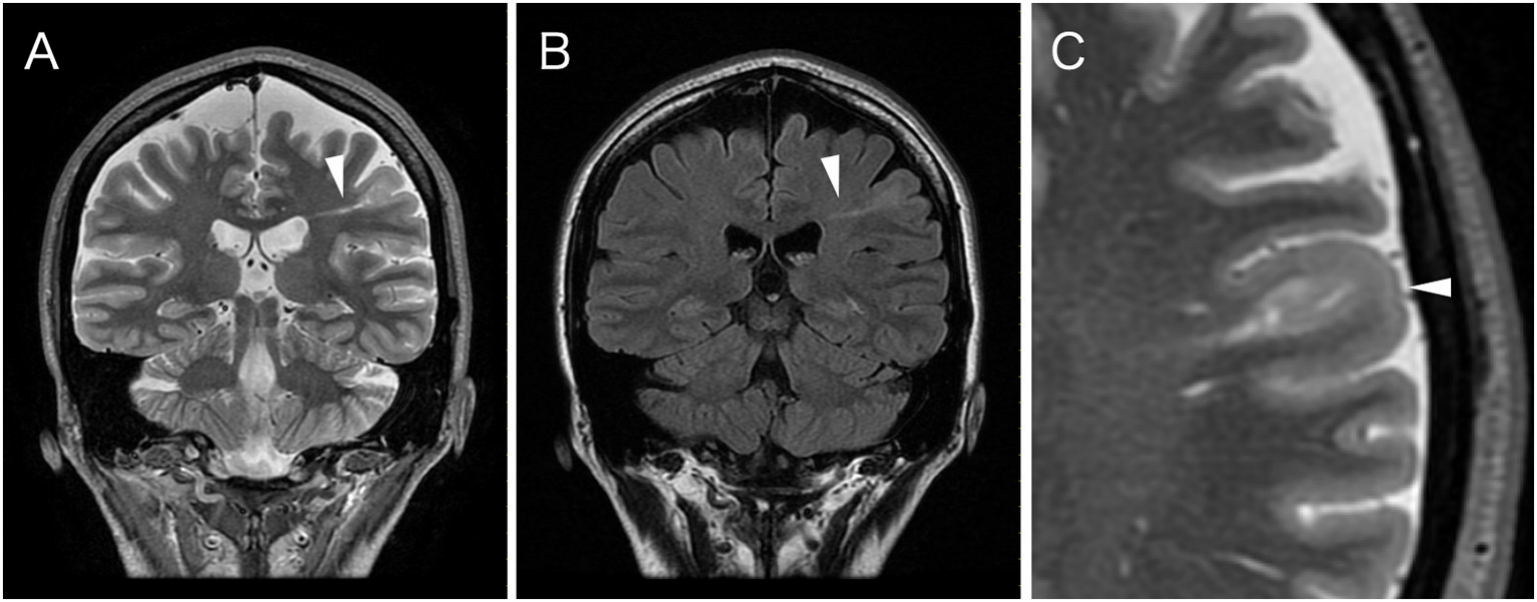
Coronal spoiled gradient recalled echo (STIR) image **(A)** and fluid attenuated inversion recovery (FLAIR) image **(B)** show an abnormal hyperintense signal band extending from the ventricle to the subcortical area (arrowhead). The signal is difficult to discern on the T1-weighted image; STIR or FLAIR images are most useful in detecting a transmantle lesion. **(C)** The magnified axial STIR image reveals cortical thickening and blurred gray/white matter junction of structurally abnormal lesion (arrowhead).

**Figure 3:**
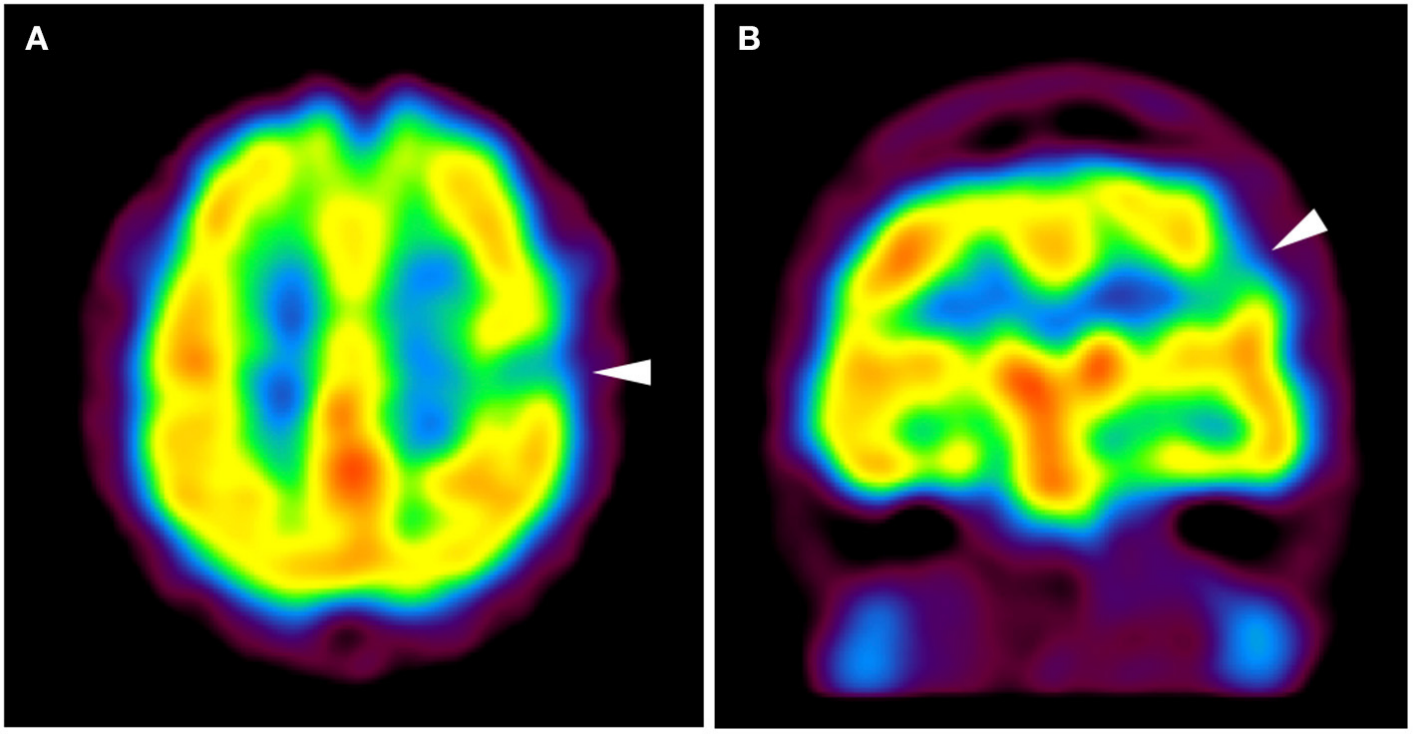
Axial **(A)** and coronal **(B)** images of technetium-99m ethyl cysteinate dimer single-photon emission computed tomography show hypoperfusion in the left frontoparietal portion (arrowhead). The area of hypoperfusion appears to be concordant with the structurally abnormal gyrus shown in Figures 1, 2.

A 3.0 Tesla MRI revealed a high-intensity area on T2-weighted/fluid attenuated inversion recovery (T2W/FLAIR) images (Figures 1A,B) and a low intensity area on spoiled gradient recalled echo images (SPGR) (Figure 1C) in the caudal portion of the left postcentral gyrus. Additionally, a high-intensity area on coronal FLAIR/short tau inversion recovery (STIR) images extended from the body of the lateral ventricle to the cortex (Figures 2A,B). STIR images showed cortical thickening and blurred gray/white matter junctions (Figure 2C). An isodense lesion was observed on a computed tomography (CT) scan, and no calcification was observed. Technetium-99m ethyl cysteinate dimer single-photon emission computed tomography showed hypoperfusion in the left frontoparietal lobe, which was consistent with the MRI lesion (Figures 3A,B).

## Clinical course

Surgical treatment was planned based on the presumptive diagnosis of type II FCD. Prior to resection, chronic subdural electrodes were implanted to record an electrocorticogram, and the extent of the epileptic focus was determined. Functional mapping using electrical cortical stimulation was also performed. Resection surgery (gyrectomy) was performed in the awake state to avoid neurological deficits. The patient recovered completely from a transient impairment of deep sensation within a month. Epileptic seizures disappeared after surgery for 6 months.

## Histological examination

The buffered-formalin fixed, paraffin-embedded sections of the resected specimen were stained with hematoxylin and eosin (H&E), Klüver–Barrera (KB), Manlow, and immunohistochemistry to which the following primary antibodies were applied: neuronal nuclei (NeuN) (A60; 1:2,000; Millipore, Burlington, MA, USA), synaptophysin (SY30; 1:500, Dako Cytomation, Glostrup, Denmark), neurofilament protein (NFP) (2F11; 1:500, Dako Cytomation, Glostrup, Denmark), nestin (Rabbit IgG, 1:200; IBL, Maebashi, Japan) and glial fibrillary acidic protein (GFAP) (GA5, 1:500; Novocastra, Newcastle upon Tyne, UK).

**Figure 4:**
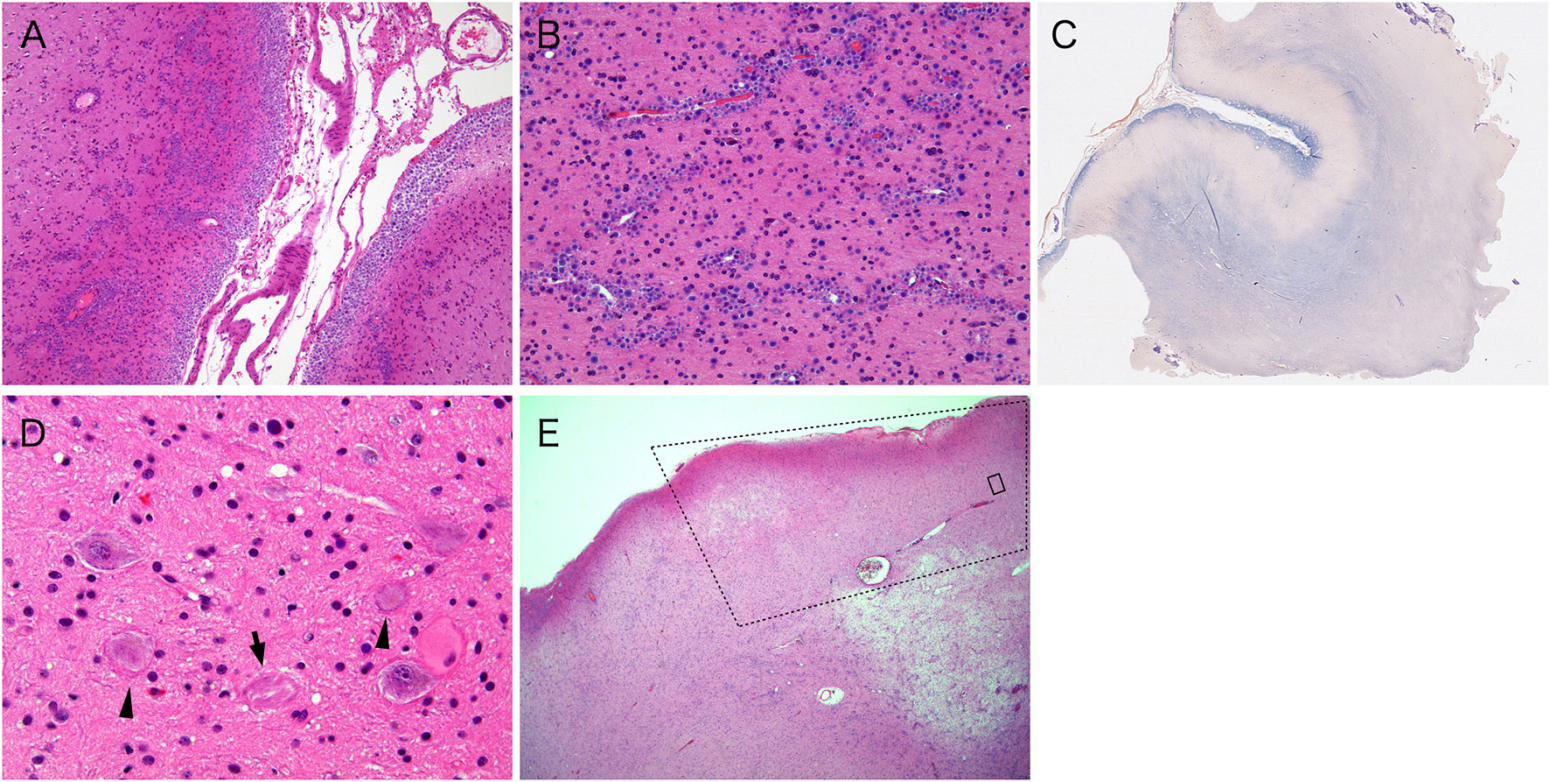
**(A)** Corpora amylacea accumulate in the subpial and the perivascular spaces in the superficial cerebral cortices (hematoxylin and eosin stain). **(B)** Corpora amylacea also accumulated in the perivascular spaces of the subcortical white matter (hematoxylin and eosin stain). Note the density of reactive astrocytes are in the background tissue. **(C)** Manlow staining highlights gliosis in the subpial cortex and the white matter in purple. **(D)** Dysplastic neurons, some of which contain neurofibrillary tangles (arrow), and a balloon cell (arrowheads) are present in the focal area of distal aspect of the resected specimen (hematoxylin and eosin stain). **(E)** The dotted square indicates the area of focal cortical dysplasia. Outside the dotted square, the cortex and subcortical white matter are abnormal, demonstrating accumulation of corpora amylacea in perivascular space (hematoxylin and eosin stain). Note that corpora amylacea does not exist within the FCD. The black square corresponds to **(D)**.

Histological examination with the H&E and KB stains revealed thickening of the superficial cortical layer of the cerebrum with abundant corpora amylacea, which accumulated in the subpial space extending along with the perivascular, Virchow–Robin space (Figure 4A). Moreover, such accumulation of corpora amylacea was also extensive in the broad areas of the subcortical white matter (Figure 4B) where the dens fibrillary gliosis highlighted by the Manlow stain (Figure 4C) and GFAP shown by immunohistochemistry was present, obscuring the cortico-medullary junction. Immunohistochemistry analyses using the neuronal markers failed to identify an abnormality in the cerebral cortical lamination or cellular dysmorphism of the cortical neurons in these gyri with thick accumulations of corpora amylacea.

In addition, at the very end of the resected specimen, a focal cortical area with disorganized cortical architecture exhibited scattered dysplastic neurons and balloon cells (Figure 4D) positive for NeuN, NFP, and synaptophysin. This feature corresponds to type IIb FCD of the International League Against Epilepsy (1). In addition, several dysplastic neurons contained neurofibrillary tangles. Moreover, there were no corpora amylacea in the subpial zone, and the perivascular space within the cortex with FCD, whereas in the subcortical white matter beneath the FCD, the corpora amylacea accumulation around the perivascular spaces were observed (Figure 4E).

## Discussion

The transmantle sign is the radiographic finding of a high-intensity area on T2W/FLAIR extending from the body of the lateral ventricle to the cortex and is highly specific to type II FCD (2–4). However, linear bands of periventricular white matter can also be seen in tuberous sclerosis complex (TSC), where a radially oriented solitary cortical tuber can closely resemble the transmantle sign (5). In 2012, the International Tuberous Sclerosis Complex Consensus Group updated the diagnostic criteria to state that cerebral white matter radial migration lines seen in TS arise from a similar pathologic process of other forms of cortical dysplasia (6). Although the exact pathogenesis is uncertain, both conditions are considered to be related to the dysfunction or injury of radial glial fibers that form the scaffolding over which neurons migrate from the periventricular germinal matrix to the cortex (2, 7). In the current case, the resected specimen contained FCD in a very limited area, and thus other factors that can cause fiber damage might have been present. One possibility is that abnormal persistent firing may have caused injury of the radial glial fibers. This patient had experienced frequent epileptic seizures for several decades. According to this theory, severe neocortex epilepsy in a patient for a long duration could show transmantle-like features.

In terms of histological examination, no FCD was identified in the majority of the resected specimen where the dense subpial cortical and white matter accumulation of the corpora amylacea were present. The resected specimen consisted of two consecutive gyruses that sandwiched the sulcus (Figure 4A) just above the subcortical white matter and showing abnormal signals on the MRI scans (3.0 × 2.6 × 1.0, 3.0 × 1.9 × 1.0 cm, respectively). Corpora amylacea were widely found in the subpial and around blood vessels of the cortical/subcortical area. On the other hand, the gyrus with cortical dysplasia, which existed next to the gyrus where the corpora amylacea accumulated, was not accompanied with corpora amylacea (Figure 4E). When comparing the resected specimen with the MRI scans, the cortex with cortical dysplasia was adjacent to the band-shaped region of the subcortical white matter showing abnormal signals but did not show high signals on STIR or FLAIR images. Based on the above information, we determined that the transmantle-like features were not due to cortical dysplasia itself, but reflected abnormal tissue in which the corpora amylacea were widely accumulated and exhibited strong gliosis.

Similar histological findings, such as those seen in neurodegenerative disorders, called Lafora body can essentially have the same biochemical components as corpora amylacea. But the Lafora bodies are considered to occur due to metabolic disorders and can appear throughout the central nervous system. On the other hand, corpora amylacea are generally considered non-specific secondary changes, that develop during ischemia or the normal aging process. They typically appear in localized areas, or multifocally throughout the subpial, subependymal, and perivascular space. Although FCD may well have caused long-standing epilepsy, a high-intensity band on T2W/FLAIR was likely to reflect an unusual accumulation of corpora amylacea in the subpial and subcortical area that was may be due to the secondary changes following intractable epilepsy (8–10).

## Conclusion

We report on a case of transmantle-like features with atypical histopathological findings. The present case suggests that the origin of a high-intensity area on T2W/FLAIR extending from the ventricle to the cortex may be related to extended accumulation of corpora amylacea. This case report supports that not all transmantle-like features are directly adjacent to a type II FCD or TSC lesions.

## Data Availability Statement

The raw data supporting the conclusions of this article will be made available by the authors, without undue reservation.

## Ethics Statement

Ethical review and approval was not required for the study on human participants in accordance with the local legislation and institutional requirements. The patients/participants provided their written informed consent for the publication of this case study. Written informed consent was obtained from the individual(s) for the publication of any potentially identifiable images or data included in this article.

## Author contributions

TM: conceptualization and surgical operation. TK: histological examination. YN: image examination. TM and SF: writing an original draft. All authors: writing—review and editing. All authors contributed to the article and approved the submitted version.

## Funding

This work was supported by MHLW Research program on rare and intractable diseases, Grant Number JPMH20FC1039.

## Conflict of interest

The authors declare that the research was conducted in the absence of any commercial or financial relationships that could be construed as a potential conflict of interest.

## Publisher's note

All claims expressed in this article are solely those of the authors and do not necessarily represent those of their affiliated organizations, or those of the publisher, the editors and the reviewers. Any product that may be evaluated in this article, or claim that may be made by its manufacturer, is not guaranteed or endorsed by the publisher.
